# A Comprehensive Collection and Analysis Model for the Drone Forensics Field

**DOI:** 10.3390/s22176486

**Published:** 2022-08-29

**Authors:** Fahad Mazaed Alotaibi, Arafat Al-Dhaqm, Yasser D. Al-Otaibi, Abdulrahman A. Alsewari

**Affiliations:** 1 Faculty of Computing and Information Technology (FCIT), King Abdulaziz University, Jeddah 22254, Saudi Arabia; 2 Faculty of Engineering, School of Computing, Universiti Teknologi Malaysia, Skudai 81310, Malaysia; 3 Department of Information Systems, Faculty of Computing and Information Technology in Rabigh, King Abdulaziz University, Jeddah 21589, Saudi Arabia; 4 School of Computing and Digital Technology, Faculty of Computing, Engineering and the Built Environment, Birmingham City University, Birmingham B4 7XG, UK

**Keywords:** drone forensics, smart cities, UAV, design science research

## Abstract

Unmanned aerial vehicles (UAVs) are adaptable and rapid mobile boards that can be applied to several purposes, especially in smart cities. These involve traffic observation, environmental monitoring, and public safety. The need to realize effective drone forensic processes has mainly been reinforced by drone-based evidence. Drone-based evidence collection and preservation entails accumulating and collecting digital evidence from the drone of the victim for subsequent analysis and presentation. Digital evidence must, however, be collected and analyzed in a forensically sound manner using the appropriate collection and analysis methodologies and tools to preserve the integrity of the evidence. For this purpose, various collection and analysis models have been proposed for drone forensics based on the existing literature; several models are inclined towards specific scenarios and drone systems. As a result, the literature lacks a suitable and standardized drone-based collection and analysis model devoid of commonalities, which can solve future problems that may arise in the drone forensics field. Therefore, this paper has three contributions: (a) studies the machine learning existing in the literature in the context of handling drone data to discover criminal actions, (b) highlights the existing forensic models proposed for drone forensics, and (c) proposes a novel comprehensive collection and analysis forensic model (CCAFM) applicable to the drone forensics field using the design science research approach. The proposed CCAFM consists of three main processes: (1) acquisition and preservation, (2) reconstruction and analysis, and (3) post-investigation process. CCAFM contextually leverages the initially proposed models herein incorporated in this study. CCAFM allows digital forensic investigators to collect, protect, rebuild, and examine volatile and nonvolatile items from the suspected drone based on scientific forensic techniques. Therefore, it enables sharing of knowledge on drone forensic investigation among practitioners working in the forensics domain.

## 1. Introduction

**Figure 1:**
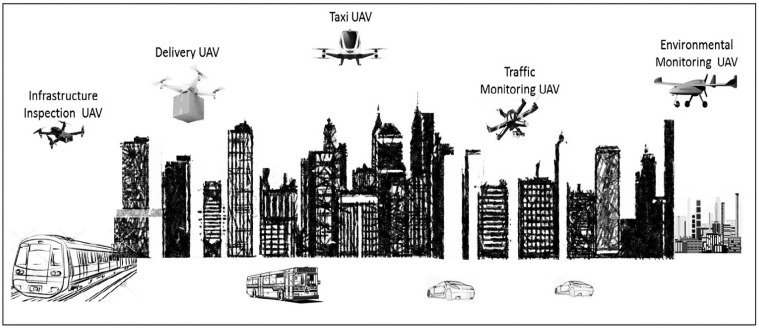
Using UAVs for various purposes in the smart environment [1].

Unmanned aerial vehicles (UAVs) are flexible and rapid mobile boards applicable to different purposes, especially in smart cities, including traffic observation, environmental monitoring, and public safety, as shown in Figure 1. UAVs have received tremendous attention since these vehicles can be controlled and monitored without pilots, based on pre-programming flight paths. In the start, this technology was adopted for military purposes, but it has been recently used even to facilitate common people’s lives.

This technology is most commonly used in area monitoring, inspection, surveillance, and cargo purposes [2,3]. It improves life quality, especially by monitoring special public events and using shared space [4]. It is observed that the airspace is loaded with a great deal of traffic, and collision accident reports are increasing day by day [5]. Due to UAVs’ traffic patterns and technology, tackling the congestion and any incident is more challenging compared to traditional networks [6,7]. The increasing trend of congestion leads to high interference, data dropping, overhead, and other abnormalities in the control system. Various types of UAVs are currently available, including small drones, aircraft, or weapon-enabled drones [8,9]; small drones fly like small insects; they can fly around an area of interest to monitor vital signs and inform the base station for further decision-making. However, the nature of data is scattered in these networks where different types of devices are involved, including routers, switches, sensor nodes, and SD cards [10,11,12]. The data are located in different places, and there is a need for proper investigation and planning to extract the forensic features. 

The drone functionalities involve several items such as the ground-station controller, onboard power management system (PMS), onboard flight control board (FCB), (multi)rotor system, electronic speed controller (ESC), and transceiver control unit (TCU) [13,14]. Regarding the potential digital forensic items, the ground-station controller, ESC, FCB, TCU, and PMS offer potentially reliable sources of evidence [15]. The information of log and memory could be taken out of the ground-station controller unit. It can be a software platform or a customized base-station design, which can interact with FCB. As drones are unmanned, TCU plays a crucial role by providing a medium for communication and control between the base station and FCB as well as a medium of communication for different sensors installed on the drone. FCB acts as the drone brain [16,17] by integrating and coordinating the information received from the functional drone units (including the mounted sensors), the inertial measurement and controls, and the flight trajectory and navigation control, and also by the management of power and being in communication with the station on the ground. Additionally, ESC is the core functionality in a drone: an electronic circuit house responsible for managing the speed and overall efficiency of a drone’s movement [18]. 

**Figure 2:**
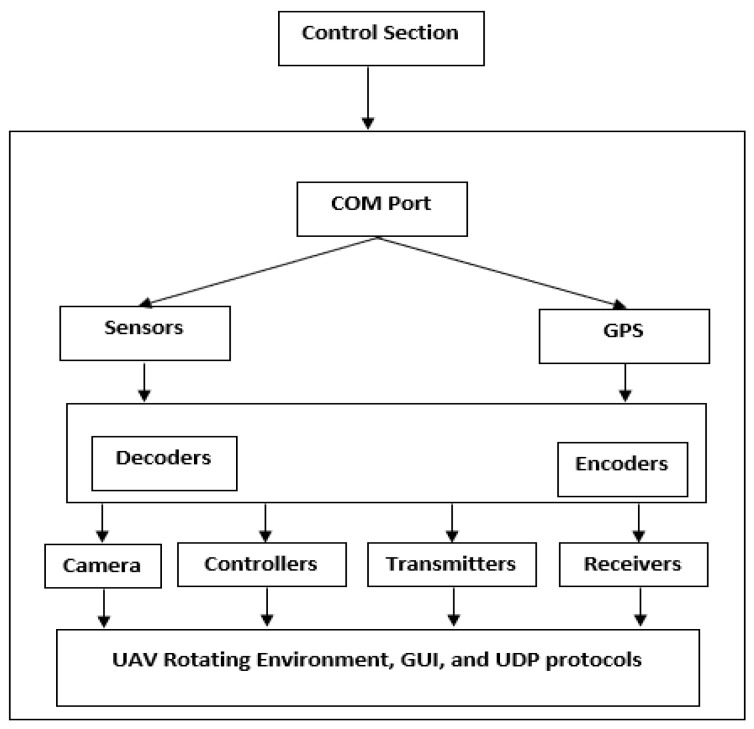
A UAV architecture composition.

Thus, drone forensic items of drone devices could be observed in the form of data stored in the memory (practical use of memory forensics), electromagnetic (EM) wave data, and contents of different log files. ESC and FCB are the potential sources of memory items, which could be directly taken out of the components of ESC and FCB, respectively. The components include flight control data, flight record data, information from the drone’s internal monitoring unit, and data from the mounted transceivers and sensors. Generally, the signal processing methodologies complement the process of forensically identifying and extracting the data. Nevertheless, the digital items, which appear in the form of EM signals from the respective transceivers and mounted sensors, can make further corroborative information available to the investigation process. In this sense, TCU could be leveraged to extract primary EM signals, which could be processed further to extract the secondary corroborative digital items. Figure 2 illustrates a UAV’s architecture and the protocols supporting its communications, which may help investigators use it during the investigation of drone crimes.

The existing literature consists of various collection and analysis models proposed for drone forensics, some of which are inclined towards specific scenarios and drone systems. As a result, there is no suitable and standardized drone-based collection and analysis model devoid of commonalities that can solve future problems that may arise in the drone forensics field. 

To address the gap mentioned above, there is a need to identify a novel comprehensive collection and analysis forensic model focusing on collection and analysis as a step toward solving redundancy. Accordingly, the present paper proposes a novel comprehensive collection and analysis forensic model (CCAFM) applicable to the drone forensics field. In comparison with the existing models, CCAFM incorporates not only the reconstruction, analysis, and collection but also the post-incident investigation processes. The processes suggested in this paper explicitly address the need for novel process models that can satisfy the requirements of post-incident response strategies during drone forensic investigations. Moreover, note that this study is contextually dependent on the initially proposed models, which have been leveraged in identifying the research gap; this will become apparent in the later sections that depict the same.

The remainder of this article is arranged as follows: Section 2 discusses the related work of the drone forensics field. Then, Section 3 concentrates on discussing the methodology adopted in this paper. After that, Section 4 gives the results and discussion. Finally, the conclusion and future remarks are provided in Section 5.

## 2. Background and Related Work

This section focuses on two parts of the drone forensics field: (1) drone forensic models and frameworks and (2) machine learning techniques used in the drone forensics field. 

### 2.1. Drone Forensic Models and Frameworks

The literature on drone forensics has been loaded with different models and frameworks proposed by various scholars. They consider four perspectives in common: forensic analysis, non-forensic analysis, forensic framework, and application in the forensic analysis [16]. For instance, in [8,9], the researchers focused on the ways to improve the evidence needed in cases where a drone is examined under digital forensics conditions. They concentrated upon the wireless forensics aspects. On the other hand, in [19], the authors discussed all components of a drone. They all emphasized the use of the Linux operating system and its potential to gather evidence on the Linux file system. Note that to work properly, drones need to use an OS. The researchers in [20] attempted to build a tool using Java-FX to visualize the real-time flight control. Their designed tool is not directly applicable to the DF field; however, it can create efficient connections between a drone and its controller to transfer data. In addition, this tool can display sensor parameters, including GPS, IMU, and altitude for pilots, providing a great level of flight safety [21,22]. In the same way, the researchers in [23] forensically examined the DJI Phantom 2 Vision Plus to find out whether the flight path of a UAV can be reconstructed using positional data collected from the UAV. They also carried out a brief examination of counter-forensic methods to discover whether the record of a flight path can be detected. In [24], the authors conducted a preliminary forensic analysis on the Parrot Bebop, known as the only UAV similar to the Parrot AR Drone 2.0. In [14], the most important challenges in UAV forensic analyses were addressed; then, two separate parts, i.e., UAV and flight controller, were investigated. In that study, the author retrieved the flight-related data from the device in the form of “.pud” files and then created a novel “.pud” file at each session between the UAV and the controller. In the case of each “.pud” file, at the opening point of the file, a set of metadata was found, comprising the UAV’s serial number, the date and time of the flight, the flight controller model, and the flight controlling application. After that, the author attempted to determine the images and videos recorded by the UAV’s onboard camera. In the images, there were the EXIF data showing information about the latitude and longitude coordinates of the sites from which the images had been taken. However, the owner of the device could be identified only if the UAV and controller are seized by determining the serial number of the device.

In [19], a general review was performed on drone forensics using the DJI Phantom 2. The breakdown analyses of the drone’s software and hardware components were conducted; then, the way the components could be used when implementing drone forensics was examined. The results obtained in that study established a belief in the persistence and scope of drone forensics. In addition, the study findings could facilitate having deeper insight into this concept and enhance its quality. Furthermore, in [25], working on the Parrot AR Drone 2.0, the authors attempted to integrate the visualizing data recovered from drones with a non-forensic approach. They designed an application to visualize the log parameters from flight data. However, only a small number of drones were evaluated in their study. The researchers in [26] analyzed the drones’ vulnerabilities and applications and their relationships with issues that generally arise in the cybersecurity domain. They asserted that if a drone is hacked and abused by opponents, serious risks or consequences may arise. That study primarily focused on identifying the benefits of using drones in numerous conditions, from employing these devices as children’s toys to using them as mass destruction weapons.

The authors in [27] proposed a 12-phase forensic framework to offer an innovative approach to the systematic investigation of UAVs. Wide-ranging tests were carried out on five commercial UAVs, for instance, the Parrot AR Drone 2.0, to identify the relationships amongst various components. They also executed an experiment to validate their developed framework. All the UAVs tested in the study were modified by adding and removing some parts. These modifications were done to check whether the framework involved all of the various elements in any basic commercial UAV and to examine whether it could be applied to a comprehensive UAV analysis. They found out that an important issue that does not allow for mitigating the attacks effectively is the deficiency of law enforcement training processes in UAVs. None of the UAVs were exposed to forensic analyses; however, an effective framework was finally constructed, which applied to the examination and analysis of the stages involved. 

The authors in [28] were the first researchers that comprehensively analyzed the DJI Phantom 3 Standard. The examined UAV was flown towards two different sites. Then, the collected data were separated into three parts: controller, drone, and phone/tablet. Eventually, they explored two types of files of interest: the “.dat” files produced by the UAV and the “.txt” files produced by the DJI GO application. The files were first subjected to the decryption and decodification processes; after that, the information about the GPS locations, flight status, Wi-Fi connections, remote control, motors, etc., was extracted. When the obtained data were analyzed, and the proprietary file structures were well-understood, the researchers developed the DROP tool for the analysis of the evidentiary files. They also developed a forensically-sound open-source drone parser (DROP) tool.

In [29], the researchers comprehensively discussed how the GPS coordinates could be used as location evidence while examining the crimes committed with the help of a drone. They attempted not only to extract the system logs but also to visualize GPS coordinates on maps, where the web-based third-party platforms were used to plot the flight paths. 

In [30], the authors explored the flight data correlation among drones, SD cards, and mobile phones. Finding a connection between a drone and a suspect significantly facilitates criminal inspections. The application of specific software to private UAV devices could provide many digital items such as GPS timestamps and waypoints, several connected satellites, barometer, pitch, roll, battery status, azimuth, distance, photos, and videos. 

In [31], the essential major log parameters of the autonomous drone were analyzed, and it was suggested to employ comprehensive software architecture related to drone forensics with preliminary results. The researchers expected that their developed software could provide a user-friendly graphical user interface (GUI) based on which the users could extract and investigate the onboard flight information. In addition, they claimed their findings would contribute to the body of the drone forensics field by designing a new tool that greatly helps run investigations effectively on criminal deeds executed with the help of drones. 

As reported in [32], open-source tools, e.g., ExifTool and CsvView, have been used in different studies to extract items from mobile applications of drones using mobile forensic techniques. The researchers in that paper used Windows and Kali (a Linux distribution) as forensic workstations to conduct the needed analyses on A.R Drone and DJI Phantom 3. Different open-source tools such as Geo-Player have been used primarily to visualize the data related to the flight path. Due to the absence of a proper built environment, including a package manager, configuration tools, and a compiler within the UAV system, this option entails making a serious change to the data existing in the UAV. Therefore, it was terminated in favor of the logical level acquisition. This was carried out by mounting a forensic mass storage device onto a UAV; the existing files were copied entirely from the mounted “/ data” partition using the “cp” command. 

Ref. [33] discussed the challenges that might arise during a UAV/drone forensic analysis. For this purpose, the currently employed forensic guidelines were evaluated for their efficiency when used in the DRF domain. After that, the authors offered their own set of guidelines in this regard. To end with, they explained how their procedures could be effectively implemented when analyzing a drone forensically. They employed DJI Phantom 3 drone as their case study. A key limitation in UAV forensics is that there is not any confirmed forensically useful tool (this indeed recommends a direction for future research). For example, the subsequent logical step is the creation of different parsing tools that can analyze original data and make available readable and reliable information. In addition, UAVs are expected to attain the capacity needed for being properly integrated with radio communication services in the future.

In [34], a novel architecture was introduced using the ID-based Signcryption to guarantee the authentication process and privacy preservation. In the initial step, the authors defined the key elements that the architecture relies on. After that, they investigated the interactions between these elements to explore how the process goes on. Next, they elaborated on their proposed authentication scheme. Thus, the RFID tags were applied to tracking the drones and the temporary identity to preserve privacy. In addition, they simulated the calculation of the average renewal of temporary identity by testing the drones’ different times and speeds. 

The researchers in [35] made a forensic analysis of a captured UAV. Security forces may capture suspected UAVs using different techniques or tools such as a shotgun; these devices may break into private properties. It is necessary to determine what software/hardware modules are used to examine a UAV. After that, the investigator needs to perform three activities: gathering accessible evidence, providing the chain of custody, and analyzing the media/artifact loaded on the UAV. The increasing incidence of unlawful utilization of UAVs reflects legal ambiguity and uncertainty in the existing aviation regulations. This problem has resulted in a shortage of evidence and fundamental standards. 

In [36], the authors attempted to identify the potential cyber-physical security threats and address the current challenges attributed to UAV security before a time in the future when UAVs are the predominant vehicles used by ordinary people. Furthermore, in that study, there is a suggestion about using a certain method that can be applied effectively to examining large-scale cyber-security attack vectors of such systems concerning four classes of systems, which are highly important to UAV operations. Furthermore, the authors elaborated on the contributions of their findings and suggested the appropriate ways to defend against such attacks. The researchers in [37] designed arbitrary software and then applied it to a locked target to gain access to the device’s interior sensors and logs with the help of neutralization and hardening strategies to predict the effectiveness. The researchers in [38] designed an innovative scheme called distributed, agent-based secure mechanism for IoD and smart grid sensors monitoring (DASMIS). They aimed to test a hybrid of peer-to-peer (P2P) and client-server (C/S) network architecture with reduced protocol overheads for immediate and bandwidth-efficient communication. Each node within this system is assigned with an initial status and provided with a python-based agent that can scan and detect in read-only node IDs, node MAC address, system calls made, node IP address, all running system programs and applications, installed applications, and modifications. The agent securely authenticates the nodes, puts communications in a coded form, and approves inter-node access. This can prevent and detect different attacks, e.g., modification, masquerading, and DoS attacks. In addition, it can execute data encryption and hashing and report the changes to other peer nodes and the server located at the C&C center. In [39], the researchers attempted to facilitate the processes such as generating, analyzing, validating, and optimizing data to trace evidence recovery. To do this, they introduced and explained the approach adopted for solving this problem considering the target fiber retrieval context using self-adhesive tapes. 

In [40], the authors attempted to adapt digital forensic processes to enhance drone incident response plans by implementing the drone forensic analysis process. The authors in that study provided more detailed information about the developed Drone Forensics and Incident Response Plan. They concluded that the Federal Aviation Administration (FAA) could update what unmanned aerial systems (UAS) require based on two classifications of UAS. In addition, they performed an inclusive review of the existing literature. They found that it lacks research concentrating on incident responses and forensic analysis frameworks designed specifically for remotely piloted aerial systems. Then, they attempted to bridge this gap. The researchers in [41] introduced the concept of “electromagnetic watermarking” as a technique exploiting the IEMI impacts to embed a watermark into civilian UAVs so that forensic tracking could be done well. In [42], many aircraft accident investigators and drone forensics investigators were surveyed to find out how they employ forensic models to carry out forensic analyses on drones. The authors analyzed the data using the chi-square test of independence; it revealed no significant connection between the drone investigations of the groups of respondents and the techniques they use to perform UAS forensics. [43] introduced a new method to accurately and quickly determine whether a drone is lying on the ground or in the sky. These results are attained just by eavesdropping on the radio traffic and processing it using standard machine learning techniques (instead of using any active approach). The authors in that study asserted that if the network traffic is classified properly, the exact status of a drone could be accurately determined using the overall operating system of ArduCopter (for instance, several DJI and Hobbyking vehicles). Furthermore, a lower bound was created on the detection delay when using the aforementioned method. It was confirmed that their proposed solution could discriminate against a drone’s state (moving or steady) with approximately 0.93 SR in 3.71 s. The researchers in [44] assessed and discussed the security vulnerabilities of Parrot Mambo FPV and Eachine E010 drones. They then suggested proper countermeasures to enhance their resilience against possible attacks. The findings showed that Parrot Mambo FPV was vulnerable to de-authentication and FTP service attacks, while Eachine E010 was susceptible to radio frequency (RF) replay and custom-made controller attacks.

The authors in [45] discussed the overall legal processes that need to be taken into action to collect drones from the crime scene and investigate them in the laboratory. In addition, in [46], a model was introduced for collecting and documenting digital data from the flight items and the related mobile devices to aid investigators in forensically examining two common drone systems, i.e., the Mavic Air and DJI Spark. Recently, several studies have been conducted in the drone forensics domain. For example, in [47], a novel drone forensic readiness framework was proposed; however, it lacked a real implementation. Moreover, the authors addressed several issues and challenges in the drone forensics domain in [16,48,49,50,51,52,53].

The variety of drone infrastructures makes drone forensics a diverse, complex, and unclear domain. This study discovered that researchers and developers typically deal with the drone forensics domain from three perspectives: drone infrastructures perspective and technical perspective as well as drone incident perspective. However, they vary in covering the perspectives. For example, some models covered all three drone forensics perspectives, whereas others covered two, and others covered only one. 

This comprehensive review of all drone forensic models reveals that the drone forensics domain lacks a unified model/framework for data collection and analysis. There is a lack of a post-investigation stage that can facilitate evaluating the investigation stage and overcoming previous mistakes. Accordingly, this study aims to develop a novel comprehensive model called a comprehensive collection and analysis forensic model to unify redundant and overlapping collection and analysis processes and activities in the drone forensics field.

### 2.2. Machine Learning Techniques Used in the Drone Forensics Field

Machine learning (ML) is an artificial intelligence (AI) area that deals with developing mathematical predictive models. These models are created in a way to analyze large volumes of data and uncover repeated patterns by using the underlying correlations among the various components of the data. This aids in the decision-making process without human interference. Such techniques also attempt to increase the forecast accuracy by learning from “experience” (also known as historical data). A training phase and a testing phase are both included in machine learning algorithms. The process of enhancing prediction performance is closely based on the process of training the model, when these models are given a large amount of historical data to produce mathematical values, simulating an artificially trained brain. Systems security [54], natural language processing [55], robotic vehicles [56], fraud detection [57], text and handwriting classification [58], object categorization [59], digital forensics [60], and speech recognition [61] are some of the areas of ML. ML models may also be applied to the discovery and detection of hidden patterns in the data being analyzed as well as the classification of the data. This is where the process is tested. Each of these algorithms follows a different approach to data analysis. Random forest, naïve Bayes, KNN, linear regression, artificial neural network (ANN), SVM, and decision tree are examples of such techniques. ML has been applied to studying a variety of issues linked to UAV. The authors in [62] presented a comprehensive study of machine learning algorithms for UAV-based communications. The study discussed how machine learning has been used to improve numerous phases of UAV-based communication, including channel modelling, resource management, positioning, and security. The paper divided the ML applications into four categories: (1) security (public safety, network jamming, and eavesdropping), (2) positioning (placement, detection, and mobility), (3) resource management (network planning, power management, routing, and data caching), and (4) physical layer (channel modelling, interference management, and spectrum allocation). The article then summarized the relevant work in each of these domains. An aggressive attempt to inject noise into a communication channel to disrupt ordinary communication exchange is known as a jamming attack. A two-classifier-based technique for identifying jamming attacks on a cloud radio access network (C-RAN) network was proposed in [63]. The multilayer perceptron (MLP) was the first classifier, and the Kernlab support vector machine (KSVM) was the second. In a low-dimensional space, jamming attacks were found to be non-linearly separable. As a result, for certain jamming attack vectors that bypass the MLP classifier, the differentiation between two classes of radio signal data can be achieved by the use of a KSVM machine learning solution. Their results were promising; they assisted to demonstrate the importance of using machine learning to classify data in order to refer to a jamming or eavesdropping attempt.

The authors in [64] proposed an anomaly detection model to reduce several attack vectors’ consequences. Their ML-based anomaly detector can detect five attack types: constant position deviation (message modification), random position deviation (message modification), velocity drift attack (message modification), DOS attack (message deletion) with constructive and destructive interference, and flight replacement attack (message injection). The automatic dependent surveillance-broadcast (ADS-B) air traffic surveillance system was the case study in their research (automatic dependent surveillance-broadcast). Preliminary ADS-B data reconstruction, combined presentation of the reconstructed and actual values to the SVDD (support vector data description) for training, and the definition and implementation of a hypersphere classifier for anomaly detection are parts of the two-step anomaly detection scheme. Reinforced learning-based power provisioning techniques are used to protect UAV transmissions from attacks such as eavesdropping and jamming [65]. ML can be used to detect an eavesdropper by building a classifier based on the received signals connected to eavesdropping attacks and non-attacks [66]. They developed the ML classifier by feeding it with data that showed a radio signal jamming attack.

Deep-learning algorithms proposed for feature extraction, planning, and situational awareness in UAV-related domains were the subject of another review article [65]. In [66], first, the researchers noted that drones frequently fly higher than typical ground user equipment. Flight altitude and line of sight propagation in open space both have an impact on radio signal transmission. They suggested a technique for locating rogue drones that could be found in a mobile network. Ground-based technology can be used to register drones that are lawful. On the other side, unregistered rogue drones that enter restricted airspace could be a security risk. The authors created virtual drone deployment scenarios for urban settings that included outside drones and ground-based equipment. The simulation scenario took into account the quantity of flying sites and sectors, inter-site distance, antennas for a base station (height and power), and carrier frequencies. Data obtained from the simulation were gathered and split into two categories: training and testing. The logistic regression (LR) and decision trees (DTs) were employed as two ML techniques. Other user equipments and drones were chosen as the two categories (variables) for LR. DT is a supervised learning model that learns by accessing feature-value tuples from a dataset. In this instance, the following items were noted: the serving cell data, the received signal strength indicator (RSSI), the standard deviation of the eight strongest reference signals, and the difference between the top two reference signals for strength. The classification results demonstrated a 100% accuracy in detecting rogue drones at heights more than 60 m and a 5% detection rate for lower altitudes. This had to deal with radio frequency interference, a more common phenomenon at lower altitudes.

Ref. [67] proposed a deep-learning-based method for detecting and identifying drones. Particular attention was paid to the identification and detection of drone acoustic fingerprints. Drones were used to create 1300 audio samples for the drone noise data standards. Additionally, to assure the accuracy of detections, the datasets included a combination of drone audio recordings recorded in an interior environment employing drone propeller sounds, stillness, and pure drone noise. To equalize audio clips, time gaps between captures were also utilized. Processing was done based on the file type, data sampling rate, and channel bitrate of each audio file. The deep-learning classifier became more successful by segmenting audio samples into more manageable portions (which were then experimented to determine the most accurate segment size). In a three-class classification experiment, the three selected classifiers—recurrent neural networks (RNN), convolutional neural networks (CNN), and convolutional recurrent neural networks (CRNN)—reported the classification of the processed drone data (drone type one, drone type two, and other noise). The CNN method was proven to produce better results than the other two.

A full drone identification approach based on ML was presented by Lee et al. in [68]. Using a CNN-based cascade classification method, the authors could classify picture data (data produced by drones with cameras) for their study. A total of 2206 drone pictures had their tags manually added. In total, 1777 were utilized for training, and the remaining 429 were used for testing. The system was able to determine the location of a drone on a camera-captured image and the vendor model of a drone based on machine classification, with stated accuracy rates of more than 90%. In [69], using the Haar feature processing method, the authors were able to extract drone sub-images with the help of the pictures collected.

The researchers in [67] offered a way to spot anomalies in a swarming flight with numerous flying drones, where the adversary might purposefully influence some drones to sabotage. Flight data from several streams were examined in order to discover these irregularities. The authors produced 16 samples per time stamp when sampling the drone data, which was made up of time-series sensory data. Prelabeled data were gathered from both normal and unusual drones. Three types of anomalies were identified: noise produced by sensor-induced signal interruptions in flight, anomalous signals generated in flight but recoverable in flight, and signal faults that force an aircraft to land as a result of a malfunction. A generative model-based 1D signal unsupervised CNN classifier was chosen for the studies.

In [68], based on the classification of drone data using ML, a drone position prediction method was defined. A naïve Bayes classifier may predict a drone’s power usage and current location using drone data gathered at the ground controller, which may allow later plans to continue or cease flying. Drone altitude, the four transmitter coils’ switching status, and the measured power transfer efficiency are among the data fields used for classification. To confirm the correctness of the classification, the resulting drone position was contrasted with the actual drone position. To create a naïve Bayes model, the classifier was trained utilizing the prior observations of the drone flight trajectory, path, and position as input. The accuracy error rates ranged from 0.09 to 45%, which were shown to be dependent on feature parameters such as transmitter coil-switching values. Based simply on the communication between the drone and the remote controller, the authors in [69] developed a methodology to detect the presence of a remotely operated drone, its current condition, and its movement. As a classifier, they used the random forest technique. It also assesses the methodology’s efficacy in the face of high packet loss and evasion attempts. The methodology was created and tested exclusively for RPAS (remotely piloted aircraft systems) drones. They showed a detection accuracy of 99.9% within 30 m without packet loss and detection accuracy of >97% within 200 m with up to 74.8% packet loss.

The authors of [70] suggested a hierarchical ensemble learning technique for radio frequency (RF) data-based UAV detection and identification. UAVs are initially detected, then their types and modes of operation are identified by the second and third classifiers. Each classifier used ensemble learning based on the KNN and XGBoost algorithms. The proposed method attained a classification accuracy of 99% with ten categories. There are three different types of UAVs, and each class indicates its nature and manner of operation (ON mode, hovering mode, flying mode, or recording mode). Additionally, in [68], the current machine-learning-based methods were examined to find a way to identify UAVs from diverse data sources.

In [71], a method was described for identifying the drone pilots via radio control signals broadcast to a UAV using a standard transmitter. Twenty trained pilots who flew the UAV on three different routes were contacted to collect the data required. There were nine characteristics in the dataset, including thrust, pitch, roll, and yaw at the time (t) and their derivatives at the time (t) (D). Additionally, a control simultaneity variable at a time (t) was provided, describing the control signals available at the time (t). The proposed system was shown to have an accuracy rate of 90% and used the random forest algorithm. The suggested method can be applied to forensic analysis in the event of a suspected drone hijacking to locate the UAV’s pilot and raise the alarm.

In [43], the authors proposed using only the encrypted communication traffic between the drone and the remote controller to determine the drone’s status (flying or at rest). A drone equipped with ArduCopter firmware was used to collect the data. Six features were produced without using the contents of the encrypted packet (inter-arrival time, packet size, mean and standard deviation computed over a certain number of samples of inter-arrival time and packet size). Three different classifiers, i.e., decision tree, random forest, and neural networks, were used to classify data. The random forest classifier yielded superior results for drone detection.

The authors of [72] recognized inter-drone communication reliability as a concern, where transmitted packets may not arrive at their intended locations. To effectively predict the transmission patterns, the authors employed ML. Utilizing a Monte Carlo simulation setup that incorporates transmission channel modeling, the success/failure probability was determined. The ML method for linear regression was combined with a comparative analysis using support vector machines (SVMs) with a quadratic kernel. The first property identified was the negative link between inter-drone distance and the likelihood of a successful packet transfer. A total of 20 drones were simulated to encourage measurement data collection. In packet transmission, the chance of communication channel success was set to 0.05. Transmission probability inside a channel, node locations, and time were all recognized as specific features for linear regression training. Quantization factor values, transmission probabilities, timings, and network node locations were among the features used by the SVM-QK classifier. The average prediction rates yielded an extremely low error rate of 0.00597.

The literature review showed that digital forensics for drones utilizing ML algorithms had received less attention. Very little research focuses on employing ML techniques for forensic analysis of drone data. The authors of [16] surveyed existing drone forensics (DRF) studies. They discussed the difficulties and possibilities in drone forensics. They also developed an approach to investigating drone-related events.

On the other hand, several models and frameworks have been proposed in the literature for drone and digital forensics to solve the challenges and issues of drone forensics [73,74,75,76,77,78,79,80,81,82]. 

## 3. Methodology for Developing and Validating CCAFM

**Figure 3:**
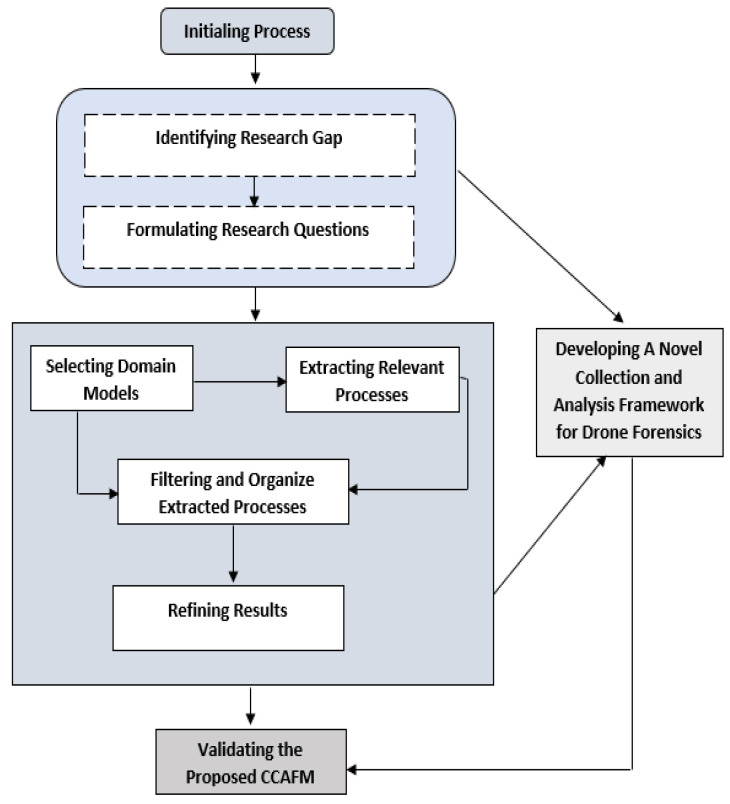
Framework for the model development process.

To validate the theoretical suppositions and achieve the main objective highlighted in this study, the current paper’s authors have systematically adapted systematic methods of design science research (DSR) for further effectiveness and efficiency. In addition, this paper aims to satisfy the need to portray the knowledge in the forensics domain. DSR was used in this study since it aids in identifying a forensic area of study, which is then sampled. The area is then mapped in order to identify pertinent and useful data that can be used to provide proof of the identified research questions. Based on the aforementioned notion, this study suggested a comprehensive forensic model for the collection and analysis processes required in the drone forensics field. Accordingly, the authors utilized a distinct criterion whose primary focus is identifying key research gaps relative to data collection and analysis in the drone forensics field. The model proposed in this study involves the following phases (see Figure 3):

1. Phase 1: Identifying and selecting the domain models
2. Phase 2: Extracting the processes for relevant investigations
3. Phase 3: Filtering and organizing the extracted processes
4. Phase 4: Proposing a comprehensive collection and analysis forensic model
5. Phase 5: Validating the proposed model

This is followed by the formulation of the research questions. Thereafter, the domain models are selected, and the relevant processes are extracted, which are then filtered and organized to aid in constructing a comprehensive forensic model for the collection and analysis. Finally, the proposed model is validated to assess its effectiveness. 

### 3.1. Phase 1: Identifying and Selecting the Domain Models

This step identified and selected the models suitable for the drone forensics investigation. Several models have been identified after reviewing the literature. Thus, the elements for identifying and selecting have been adapted from the previous research [83,84]. To identify and select the drone forensic models from the literature, authors need to employ some factors/criteria for this purpose. The highly applicable processes of drone forensic investigation processes should be widely covered based on the objective of the development process. A coverage metric indicates the applicability of the sourced model. Table 1 shows 32 common models that could be used as the output of this step.

**Table 1:** Recognized and nominated drone forensics models.

ID.	Models References	Year	Authors	Description of the Model
1.	[20]	2015	Mhatre et al.	They offered a tool using Java-FX to visualize the real-time flight control. Their designed tool is not directly applicable to the DF field; however, it can create efficient connections between a drone and its controller to transfer data. In addition, this tool can display sensor parameters, including GPS, IMU, and altitude for pilots, which provides a great level of safety for flights
2.	[24]	2016	Horsman	This study offered an initial assessment of UAV devices, emphasizing the issues caused by this equipment to the digital forensic experts and law enforcement examinations. They provided an investigation of a Parrot Bebop drone and a study of the mobile tools used to pilot it, namely Galaxy and an iPhone 6, both using the Parrot’s offered UAVs flight routing product “FreeFlight3”.
3.	[26]	2016	Mohan	In this study, the author analyzed the drones’ vulnerabilities and applications and their relationships with issues that generally arise in the cybersecurity domain. They asserted that if a drone is hacked and abused by opponents, serious risks or consequences may arise. That study primarily focused on identifying the benefits of using drones in numerous conditions, from employing these devices as children’s toys to using them as mass destruction weapons.
4.	[19]	2016	Kovar, Dominguez, and Murph	The authors discussed all components of a drone. They all emphasized the use of the Linux operating system and its potential to gather evidence on the Linux file system. Note that to work properly, drones need to use an OS.
5.	[85]	2016	Maarse et al.	Researchers tried to evaluate DRFs with the purpose of DJI Phantom 2 commonly. They assessed the drone’s software and hardware and reviewed how they can be used to apply DRFs. Their results achieved the creation of a principle in the dedication and range of DRFs
6.	[25]	2016	Procházka	Authors attempted to integrate the visualizing data recovered from drones with a non-forensic approach. They designed an application to visualize the log parameters from flight data. However, only a small number of drones were evaluated in their study. The scope of this working on the Parrot AR Drone 2.0.
7.	[29]	2017	Prastya, Riadi, and Luthfi	The researchers comprehensively discussed how the GPS coordinates could be used as location evidence while examining the crimes committed with the help of a drone. They attempted not only to extract the system logs but also to visualize GPS coordinates on maps, where the web-based third-party platforms were used to plot the flight paths.
8.	[27]	2017	Jain, Rogers, and Matson	The authors proposed a 12-phase forensic framework to offer an innovative approach to the systematic investigation of UAVs. Wide-ranging tests were carried out on five commercial UAVs, for instance, the Parrot AR Drone 2.0, to identify the relationships amongst various components. They also executed an experiment to validate their developed framework. All the UAVs tested in the study were modified by adding and removing some parts. These modifications were done to try to check whether the framework involved all of the various elements in any basic commercial UAV and to examine whether it could be applied to a comprehensive UAV analysis. They found out that an important issue that does not allow for mitigating the attacks effectively is the deficiency of law enforcement training processes in UAVs. None of the UAVs were exposed to forensic analyses; however, an effective framework was finally constructed, which applied to the ex-amination and analysis of the stages involved.
9.	[28]	2017	Clark et al.	In this study, the authors were the first researchers that comprehensively analyzed the DJI Phantom 3 Standard. The examined UAV was flown towards two different sites. Then, the collected data were separated into three parts: controller, drone, and phone/tablet. Eventually, they explored two types of files of interest: the “.dat” files produced by the UAV and the “.txt” files produced by the DJI GO application. The files were first subjected to the decryption and decodification processes; after that, the information about the GPS locations, flight status, Wi-Fi connections, remote control, motors, etc., was extracted. When the obtained data were analyzed, and the proprietary file structures were well-understood, the researchers developed the DROP tool for the analysis of the evidentiary files. Additionally, they developed a forensically sound open-source drone open-source parser (DROP) tool.
10.	[86]	2017	Bucknell and Bassindal	The authors analyzed the effects of a quadcopter’s downwash to know whether it can affect the retaining of material evidence in crime events.
11.	[30]	2017	Llewellyn	The authors attempted to explore the flight data correlation among drones, SD cards, and mobile phones. Finding a connection between a drone and a suspect significantly facilitates criminal inspections. The application of specific software to private UAV devices could lead to the provision of many digital items such as GPS timestamps and waypoints, several connected satellites, barometer, pitch, roll, battery status, azimuth, distance, photos, and videos.
12.	[32]	2017	Barton and Azhar	The researchers in this study used Windows and Kali (a Linux distribution) as forensic workstations to conduct the needed analyses on A.R Drone and DJI Phantom 3. Different open-source tools such as Geo-Player have been used primarily to visualize the data related to the flight path. Due to the absence of a proper built environment, including a package manager, configuration tools, and a compiler within the UAV system, this option entails making a serious change to the data existing in the UAV. For that reason, it was terminated in favor of the logical level acquisition. This was carried out by mounting a forensic mass storage device onto a UAV; then, the existing files were copied entirely from the mounted “/ data” partition using the “cp” command.
13.	[31]	2017	A. L. P. S. Renduchintala, Albehadili, and Javaid	The authors tried to examine the key log boundaries of the independent UAV. They suggested comprehensive forensic software for the drone design with initial findings.
14.	[87]	2018	Bouafif et al.	In this study, the authors utilized digital forensics to the Parrot A.R Drone 2.0. They delivered a dialogue on various common statements and file structures and then tried to imagine the trip path with the aid of Google Earth.
15.	[23]	2018	Roder, Choo, and Le-Khac	The authors presented a set of rules for drone examinations in this study. They tried to discern the direction to conduct a drone forensic examination with the purpose of the DJI Phantom 3 drone as a real scenario.
16.	[33]	2018	Maune	The authors offered their own set of guidelines in this regard. To end with, they explained how their procedures could be effectively implemented when analyzing a drone forensically. They employed DJI Phantom 3 drone as their case study. A key limitation in UAV forensics is that there is not any confirmed forensically useful tool (this indeed recommends a direction for future research). For example, the subsequent logical step is the creation of different parsing tools that can analyze original data and make available readable and reliable information. In addition, UAVs are expected to attain the capacity needed for being properly integrated with radio communication services in the future.
17.	[34]	2018	Benzarti, Triki, and Korbaa	In this study, a novel architecture was introduced using the ID-based Signcryption to guarantee the authentication process and privacy preservation. In the initial step, the authors defined the key elements that the architecture relies on. After that, they investigated the interactions between these elements to explore how the process goes on. Next, they elaborated on their proposed authentication scheme. Thus, the RFID tags were applied to tracking the drones and the temporary identity to preserve privacy. In addition, they simulated the calculation of the average renewal of temporary identity by testing the drones’ different times and speeds.
18.	[88]	2018	Gülataş and Baktır	The essential major log parameters of the autonomous drone were analyzed, and it was suggested to employ comprehensive software architecture related to drone forensics with preliminary results. The researchers expected that their developed software could provide a user-friendly graphical user interface (GUI) based on which the users could extract and investigate the onboard flight information. In addition, they claimed their findings would contribute to the body of the drone forensics field by designing a new tool that greatly helps run investigations effectively on criminal deeds executed with the help of drones.
19.	[36]	2018	Dawam, Feng, and Li	The authors attempted to identify the potential cyber-physical security threats and address the current challenges attributed to UAV security before a time in the future when UAVs are the predominant vehicles used by ordinary people. Furthermore, in that study, there is a suggestion about using a certain method that can be applied effectively to examining large-scale cyber-security attack vectors of such systems concerning four classes of systems, which are highly important to UAV operations. Furthermore, the authors elaborated on the contributions of their findings and suggested the appropriate ways to defend against such attacks.
20.	[37]	2018	Esteves, Cottais, and Kasmi	In this paper, authors designed arbitrary software and then applied it to a locked target to gain access to the device’s interior sensors and logs with the help of neutralization and hardening strategies to predict the effectiveness.
21.	[89]	2018	Shi et al.	The authors discussed the overall legal processes that need to be taken into action to collect drones from the crime scene and investigate them in the laboratory.
22.	[90]	2018	Guvenc et al.	In this study, a model was introduced for collecting and documenting digital data from the flight items and the related mobile devices to aid investigators in forensically examining two common drone systems, i.e., the Mavic Air and DJI Spark.
23.	[91]	2018	Ding et al.	The authors conducted a preliminary forensic analysis on the Parrot Bebop, known as the only UAV similar to the Parrot AR Drone 2.0.
24.	[35]	2019	A. Renduchintala et al.	The researchers made a forensic analysis of a captured UAV. Security forces may capture suspected UAVs using different techniques or tools such as a shotgun; these devices may break into private properties. It is necessary to determine what software/hardware modules are used to examine a UAV. After that, the investigator needs to perform three activities: gathering accessible evidence, providing the chain of custody, and analyzing the media/artifact loaded on the UAV. The increasing incidence of unlawful utilization of UAVs reflects legal ambiguity and uncertainty in the existing aviation regulations. This problem has resulted in the shortage of evidence and fundamental standards.
25.	[38]	2019	Fitwi, Chen, and Zhou	The researchers designed an innovative scheme called distributed, agent-based secure mechanism for IoD and smart grid sensors monitoring (DASMIS). Their aim was to test a hybrid of peer-to-peer (P2P) and client-server (C/S) network architecture with reduced protocol overheads for immediate and bandwidth-efficient communication. Each node within this system is assigned with an initial status and provided with a python-based agent that can scan and detect in read-only node-IDs, node MAC address, system calls made, node IP address, all running system programs and applications, installed applications, and modifications. The agent securely authenticates the nodes, puts communications in a coded form, and approves inter-node access. This can prevent and detect different attacks, e.g., modification, masquerading, and DoS attacks. In addition, it can execute data encryption and hashing, and it reports the changes to other peer nodes and the server that is located at the C&C center.
26.	[39]	2019	Jones, Gwinnett, and Jackson	The authors attempted to facilitate the processes such as generating, analyzing, validating, and optimizing data to trace evidence recovery. To do this, they introduced and explained the approach adopted for solving this problem considering the target fiber retrieval context using self-adhesive tapes.
27.	[40]	2019	Salamh and Rogers	The authors attempted to adapt digital forensic processes to enhance drone incident response plans by implementing the drone forensic analysis process. The authors in that study provided more detailed information about the developed Drone Forensics and Incident Response Plan. They resulted in the fact that the Federal Aviation Administration (FAA) can update what unmanned aerial systems (UAS) require based on two classifications of UAS. In addition, they performed an inclusive review of the existing literature. They found out that it lacks research concentrating on incident responses and forensic analysis frameworks designed specifically for remotely piloted aerial systems. Then, they attempted to bridge this gap.
28.	[41]	2019	Esteves	The researchers introduced the concept of “electromagnetic watermarking” as a technique exploiting the IEMI impacts to embed a watermark into civilian UAVs so that forensic tracking could be done well.
29.	[42]	2019	J. L. Esteves, E. Cottais, and C. Kasmi	In this study, many aircraft accident investigators and drone forensics investigators were surveyed to find out how they employ forensic models to carry out forensic analyses on drones. The authors analyzed the data using the chi-square test of independence; it revealed no significant connection between the drone investigations of the groups of respondents and the techniques they use to perform UAS forensics.
30.	[92]	2019	Le Roy et al.	A new method was introduced to accurately and quickly determine whether a drone is lying on the ground or in the sky. These results are attained just by eavesdropping on the radio traffic and processing it using standard machine learning techniques (instead of using any active approach). The authors in that study asserted that if the network traffic is classified properly, the exact status of a drone could be accurately determined using the overall operating system of ArduCopter (for instance, several DJI and Hobbyking vehicles). Furthermore, a lower bound was created on the detection delay when using the aforementioned method. It was confirmed that their proposed solution could discriminate against a drone’s state (moving or steady) with approximately 0.93 SR in 3.71 s.
31.	[43]	2019	Sciancalepore et al.	The authors proposed using only the encrypted communication traffic between the drone and the remote controller to determine the drone’s status (flying or at rest). A drone equipped with ArduCopter firmware was used to collect the data. Without using the contents of the encrypted packet, six features were produced (inter-arrival time, packet size, mean and standard deviation computed over a certain number of samples of inter-arrival time and packet size). Three different classifiers, i.e., decision tree, random forest, and neural networks, were used to classify data (decision tree, random forest, and neural networks). The random forest classifier yielded superior results for drone detection.
32.	[44]	2020	Lakew Yihunie, Singh, and Bhatia	The researchers assessed and discussed the security vulnerabilities of Parrot Mambo FPV and Eachine E010 drones. They then suggested proper countermeasures to enhance their resilience against possible attacks. The findings showed that Parrot Mambo FPV was vulnerable to de-authentication and FTP service attacks, while Eachine E010 was susceptible to radio frequency (RF) replay and custom-made controller attacks.

### 3.2. Phase 2: Extracting the Processes for Relevant Investigations

The criteria adapted from [93,94] were used to extract the processes of collection and analysis from the 32 extracted models through the following criteria:

1. Abstracts, related work, conclusions, and titles were excluded. The investigation process was extracted either from the main textual model or the diagram.
2. The investigation process should be associated with the analysis and collection processes.
3. There should be an activity, definition, or task in the investigation process with which the purpose and meaning of the process can be recognized.
4. The investigation processes unrelated to the conducting processes of collection and analysis were excluded.
5. The implicit and explicit investigation processes should be included in the models.

Table 2 shows that the 32 models consisted of 42 processes. Due to the redundancy of the majority of these 42 processes, they needed to be merged into a unified model. In the next section, this merging process is discussed.

### 3.3. Phase 3: Filtering and Organizing the Extracted Processes

This phase filters and organizes the extracted processes based on the same semantic meanings. In the first 21 processes, the drone forensics field was discussed from the perspective of preservation and collection, while the drone forensics field was discussed in the second 21 processes from the perspective of analysis and reconstruction, as Table 3 and Table 4 show, respectively.

### 3.4. Phase 4: Proposing a Comprehensive Collection and Analysis Forensic Model

**Figure 4:**
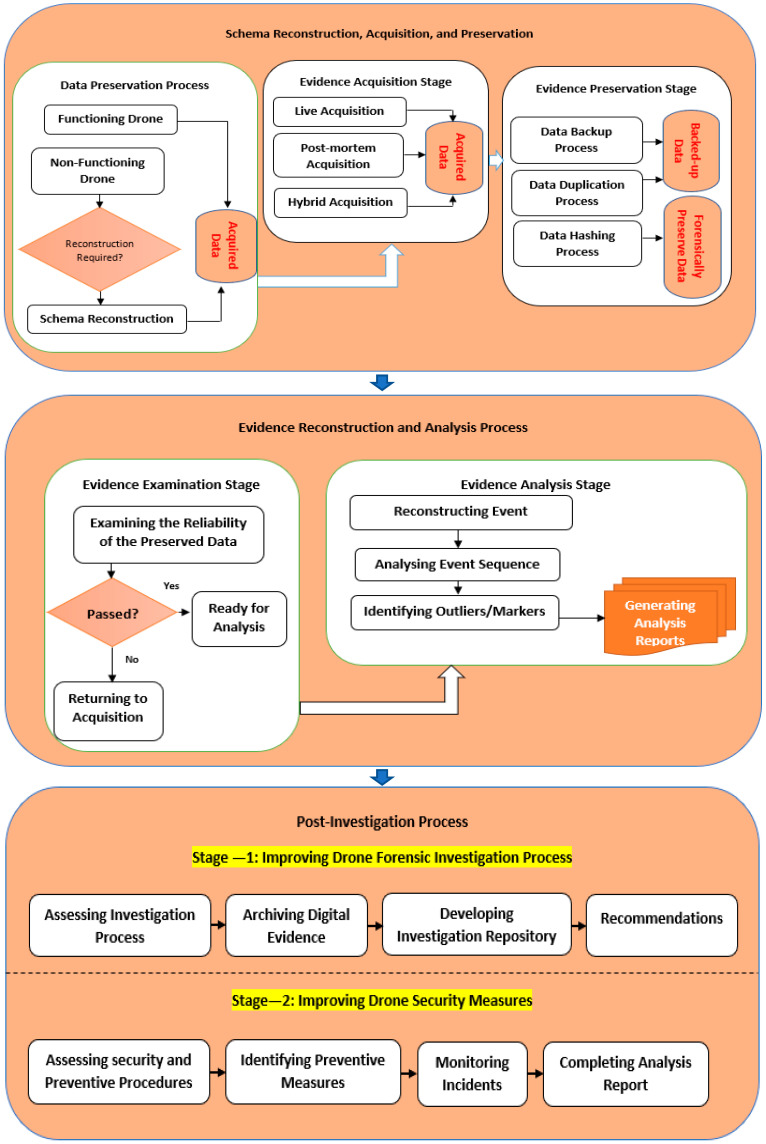
Comprehensive collection and analysis model for the drone forensics field.

Due to the overlapping of some extracted processes, the tasks and activities performed in each of the investigative processes should have been considered, and there should not be a focus on naming conventions [22,23]. A more frequent process of investigation is selected by adapting the mapping process. The proposed forensic model is shown in Figure 4. It has three common investigation processes, i.e., collection and preservation, reconstruction and analysis, and post-analysis process. The extracted 21 collection processes were covered by the collection and preservation process, while the extracted 21 analysis and reconstruction processes were covered by the reconstruction and analysis process. The post-investigation process presented as a new process has not been stated in any of the existing models of drone forensics. Its two main goals are (1) the assessment and improvement of the current investigation processes based on a group of procedures that Section 5 highlighted and (2) the assessment and improvement of the existing drone security measures to prevent further drone incidents in the future, as indicated in Section 5.

### 3.5. Phase 5: Validating the Proposed Comprehensive Collection Analysis and Forensic Model

In this phase, the focus was on validating if the proposed CCAFM was completed comparing to the available models of analysis and collection in the drone forensics field. To finalize this process, a comparison was made between CCAFM and the other models [24]. The comparison was to validate if the developed CCAFM is efficient and whether it can explain and fit into the existing models in this domain. Table 5 shows the models used in the comparison—for example, the extracted processes presented in Table 2 were compared with the proposed CCAFM processes. CCAFM was found more inclusive, encompassing the actions discovered in the former models. Figure 4 indicates that CCAFM involves three common investigation processes, i.e., reconstruction and analysis, collection and preservation, and post-investigation process.

Consequently, the redundant investigation processes of the analysis and collection processes in the proposed model are addressed given that new procedures and processes have also been proposed. The main reason for this is to improve the analysis and collection process in the drone forensics field. Table 4 shows that all the activities of the previous models have been covered in the first two processes of the proposed investigation model. For example, the first investigation process, which is the preservation and collection process, performs the entire investigation activities and the tasks of the 21 investigation processes. In addition, Table 5 shows that most of the tasks and activities of the 21 investigation processes are covered in the reconstruction and analysis process in the second process of the proposed model.

## 4. Discussion

In this study, a novel compressive collection and analysis forensic model referred to as CCAFM was proposed to be applied to the drone forensics field. The proposed model further discusses the drone forensics from two perspectives: the harmonization perspective and awareness perspective. The harmonization perspective discusses the existing redundant steps and activities used within the collection and analysis process in the drone forensics field. Thus, the current drone forensic collection and analysis processes have been grouped, combined, and harmonized in two main abstract processes based on the similarities in the meaning and functioning. Thus, the redundancy in the investigation process has caused ambiguity and heterogeneity among practitioners in this domain. On the other hand, there are several limitations of the proposed CCAFM; for example, the data used for development and validation were collected by the authors themselves. Moreover, the proposed model is validated from the completeness perspective using a comparison with another models method; it lacks real implementation to evaluate its applicability. 

### 4.1. Preservative A: Harmonized Perspective

The harmonized perspective consists of two common investigation processes:

1. Acquisition and preservation process: It allows volatile and nonvolatile items to be collected from the suspect drone and preserved based on the trusted forensic techniques mainly using the collection and preservation process. It has three main stages: schema reconstruction, data acquisition, and data preservation. Prior to attempting to conduct any investigation, the investigator would need to ascertain the state of the database. In this regard, the preparatory process can be evoked. In this process, the investigator would need to verify if the drone would require a schema reconstruction process. Drone schema reconstruction is a major technical process implemented when the drone structure is either corrupted, damaged, or altered in such a way that the functionality of the drone cannot be ascertained. In such conditions, schema reconstruction techniques will be required to re-align the raw data in the drone to the metadata of the database. This could be techniques against localized data alteration, alteration to the sequence of blocks of data, and manipulation carried out on the links between blocks of data [82,95,96]. Upon an accurate schema reconstruction, the process of evidence acquisition can be evoked. Volatile and nonvolatile data can be acquired using the data acquisition stage with trusted forensic tools. It has three stages: a dead acquisition stage, a hybrid acquisition stage, and a live acquisition stage. The dead acquisition stage means that data are copied from the system investigated without the system itself, while in the hybrid acquisition stage, key elements of both dead and live acquisition methods are combined to offer the advantages of both options. The live acquisition stage is preferred to prevent the missing of volatile data. In the live acquisition stage, data are acquired in the case of system analysis while the investigation is being done. It is a usual dead acquisition after the live acquisition of the volatile data. Acquired data are the output of this stage. In the acquired data, preservation is needed to protect integrity and confidentiality against tampering. Hashing and backup methods are used in the data preservation stage to keep the reliability of the acquired data, and any modification of acquired data could be prevented. The integrity of data can be kept by hashing and backing up the acquired data from the data acquisition stage. Hashing ensures that the data will not be changed by the drone forensics techniques used to hash the acquired data. It also assures that the transferred acquired data between the destination and source is reliable. Moreover, an exact copy of the acquired data utilized as a second copy for the tampered original data is provided in the backup. Therefore, reconstruction and analysis activities are conducted to transfer the copy of the hashed acquired data to the forensic workstation through secure channels.
2. Analysis and reconstruction: In this phase, the events causing an incident to acquired data (including user drone activities, existing SQL execution history, and retracing past systems) are examined, constructed, and analyzed. There are two stages in this mode: data examination and data reconstruction. In the data examination stage, it is ensured that acquired data are not altered and remain authentic. Thus, the investigation team’s first mission is to use such examination tools to examine if the data collected are authentic. However, the modification of the acquired data requires the investigation team to bring another clean acquired data from the first acquired data.

Then, in the data reconstruction stage, timeline events (involving user drone activities, stored procedures, function execution, past SQL execution history, and retracing past system) are reconstructed from the acquired nonvolatile and volatile data. Forensic techniques and algorithms such as LogMiner and Dragon are used by the reconstruction team (analyzers or examiners) to perform a reconstruction process. For the reconstruction process, there is a need for a clean drone environment and acquired data to construct the timeline. The timeline as a collection of digital events is recognized from the reconstruction process used in the analysis. As the digital events are recognized, the malicious drone events, successful login events, and failed login events can be identified and added to a timeline examination. Furthermore, an investigator can gain insight into the occurring events and the involved people by creating a timeline of events. There is an association between the timeline class and the forensic technique used to search and filter the timeline for giving the pieces of evidence. The drone files recorded on storage devices and hard drives are usually recognized as evidence. Its binary transmission may be relied on in a court. It involves why, how, what, when, and where the malicious transactions have been carried out. In the end, the whole data reconstruction and analysis processes are documented by the investigation team in several reports and submitted to the court and the respective company.

### 4.2. Preservative B: Awareness Perspective

It is mainly a post-investigation process: The primary focus of the post-investigation process is on collecting the data from the two previous processes, i.e., collection and analysis processes, for training, learning, and improvement purposes, generally taking the form of recommendations. These recommendations will be used as awareness forms and guidelines to improve the drone investigation in the future or prevent drone incidents in the future. It comprises two main stages:

1. Improving the drone investigation process: the investigation process can be improved in the future, and the output and reliability of the drone investigation can be enhanced by gathering the output of the preservation and collection processes and analyses as well as the reconstruction process. It includes a set of procedures:
  - Assessing the process of investigation: A reliable investigation can be guaranteed by carefully examining the existing drone forensics’ workstation, i.e., investigation team, tools, procedures, guidelines, models, methods, labs, and policies. The investigation team should have enough qualifications and high experience to perform the stages of the investigation. To preserve high productivity, the investigation team needs to be continuously trained and updated. The center of any investigation is the forensic tools, which should be carefully selected by senior investigators. The selection of improper investigation tools may result in the failed stages of the investigation and lost evidence. A reliable and clear investigation can be assumed by assessing and updating the existing investigation policies, guidelines, and procedures.
  - Archiving the digital pieces of evidence: All the incident-related data will be deleted with the exemption of digital evidence that will be securely archived for further legal purposes.
  - Developing the investigation repository: All the investigation stages are kept with an investigation repository, allowing practitioners to quickly access the previous investigations information and use it again for similar cases.
  - Recommendations: The weakness of the existing forensic workstations can be improved by translating the assessment at step 1 to new guidelines.
2. Improving the drone security measures: There is a need for enabled and audited security measures, such as access control for the mitigation of the drone breaches. This stage aims to improve the security measures for the prevention of drone incidents. It involves several procedures:
  - Assessing preventive security procedures: The integrity, confidentiality, and availability of the information systems are protected based on the security measures as significant components. To accomplish the security objectives, it is necessary to continuously assess the preventive security procedures. Thus, the data should be kept secure by reassessing and improving the current drone security measures such as IDS, access control, auditing feature, and firewalls. On the other hand, all CCAFM processes are subject to the laws of countries in terms of privacy when acquiring information. In fact, this is one of the biggest challenges facing investigators. Some laws do not allow data to be collected without permission from the authorities. For example, there is a need to strike a balance between what is acceptable for private use and what is not. Currently, there are laws being created by both state and federal agencies. Many of these laws revolve around the first and fourth amendments. In particular, the need to know the location of the drone when it is recording is critical for privacy. Some states are simply banning them outright, while others are more focused on what is allowed and what is not. One example is laws against drones carrying weapons. The state of Connecticut, USA, proposed a law to restrict/prohibit putting guns on drones after a man posted a video of his drone flying with a gun and shooting. Thus, all the processes of the proposed CCAFM should follow the laws of the state where the crime happened in terms of privacy.
  - Identifying preventive measures: It may prove dangerous someday to see no preventive measures in the drone system for monitoring outsider or insider attacks. Thus, preventive drone measures must be enabled and assessed continuously to keep data secure.
  - Monitoring incidents: The post-incident activities have been closely monitored since the reappearance of threat actors. We suggest that a security log hawk should analyze the security information and event monitoring (SIEM) data to see any signs of indicators tripping associated with the previous incident.
  - Completing an analysis report: The incident will be documented to augment additional security measures and improve the incident response plan to prevent such security events in the future. The report needs to answer the following questions: how logging is done, what are the intrusion detection systems (IDS), what is logged, and what are the forensic acquisition, reconstruction, preparation, and analyzing methods, and tools.

New processes for conducting drone investigation are included in the proposed CCAFM to fill the gap discussed in Section 2. There are no similar factors in the available drone forensic models based on the processes required to improve and assess the drone forensic investigation processes of forensic investigation of drones and drone security measures.

## 5. Conclusions

Due to the growing use of unmanned aerial vehicles (UAVs) in several applications and environments, the incidents related to these vehicles are growing rapidly. Thus, the drone forensics field is required to capture and analyze the drone crimes. Drone incidents can be collected and analyzed with different specific drone forensic models. Therefore, a novel comprehensive forensic model was proposed in this study for the collection and analysis of the data collected through the investigations in the drone forensics field. The redundancy in the examination process, which causes the ambiguity and heterogeneity among domain forensic practitioners, has been solved in the current paper by combining all the collection and analysis models existing in the drone forensics field. More specifically, the exiting processes, activities, concepts, tasks, and practices of the models were grouped and combined into a single model called CCAFM.

Furthermore, the post-investigation process was suggested in this study as a completely new investigation process. It was essentially appropriate to use a design science approach to achieve the objective of this study, i.e., developing a comprehensive model applicable to drone forensics. Three interdependent processes, i.e., schema reconstruction, acquisition and preservation, evidence reconstruction and analysis, and post-investigation, were involved in the developed model. The proposed model was compared with other models previously proposed in this field to evaluate its completeness and effectiveness. The proposed model allows domain users to gather, protect, rebuild, and examine both volatile and nonvolatile data from suspect drones using trusted forensic tools. Furthermore, it could be used as a guide for improving the drone forensic processes and drone security procedures. The future research in this domain may include the real implementation of CCAFM to verify the efficiency of the proposed model in terms of capturing, preserving, and analyzing drone incidents.

## Figures and Tables

**Table 2:** Extracted processes.

No	Similar Processes
1.	Collecting Drone Data
2.	Drone Evidence Collection
3.	Collection Drone Process
4.	Drone Items Collection
5.	Drone Data Extraction Process
6.	Starting of Investigation
7.	Drone Metadata Gathering
8.	Drone Data Collection
9.	Collecting Drone Files
10.	Drone Item Collection
11.	Collection process
12.	Collection of Drone Nonvolatile Items
13.	Collection of Drone Volatile Items
14.	Drone Item Collection
15.	Collection Suspect Drone System
16.	Drone Collection and Preservation Process
17.	Collection of Drone Process
18.	Execution of Drone Data
19.	Collection, Preservation
20.	Drone Item Collection
21.	Drone Items Collection
22.	Reconstructing Drone Events
23.	Restoring Drone Integrity
24.	Drone Media Analysis
25.	Timeline Creation of Drone Data
26.	Drone Data Recovery
27.	Search String
28.	Drone Artifact Analysis
29.	Financial and Business Data Analysis
30.	Drone Restoration and Searchability
31.	Investigation on Drone Data Collected
32.	Drone Artifact Analysis
33.	Rebuilding of Drone Data
34.	Reconstruction of Drone Events
35.	Drone Forensic Analysis
36.	Analysis of Anti-forensic Drone Attacks, Analysis of Drone Attack
37.	Reconstructing Drone Evidence
38.	Reconstruction Process
39.	Analysis Process
40.	Reconstructing Drone Volatile Items
41.	Recovering Drone Schema
42.	Analysis of Drone Stages

**Table 3:** Collection and preservation processes.

No	Similar Processes
1.	Collecting Drone Data
2.	Drone Evidence Collection
3.	Drone Collection process
4.	Drone Item Collection
5.	Drone Data Extraction Process
6.	Starting the Investigation
7.	Drone Metadata Gathering
8.	Drone Data Collection
9.	Collecting Drone Files
10.	Drone Item Collection
11.	Drone Collection Process
12.	Collection of Drone Nonvolatile Items
13.	Collection of Drone Volatile Items
14.	Drone Items Collection
15.	Collection Suspect Drone System
16.	Collection and Preservation
17.	Drone Collection process
18.	Execution of Drone Data
19.	Collection, Preservation
20.	Drone Items Collection
21.	Drone Items Collection

**Table 4:** Reconstruction and analysis processes.

No	Similar Processes
1	Reconstructing Drone Data
2	Restoring Drone Integrity
3	Drone Media Analysis
4	Timeline Creation of Drone Data
5	Drone Data Recovery
6	Search String
7	Drone Artifact Analysis
8	Financial and Business Data Analysis
9	Drone Restoration and Searchability
10	Investigation on Drone Data Collected
11	Drone Artifact Analysis
12	Reconstruction of Drone Data
13	Reconstruction of the Drone Events
14	Drone Forensic Analysis
15	Analysis of Anti-forensic Attacks, Analysis of Drone Attacks
16	Reconstructing Drone Evidence
17	Reconstruction Process
18	Drone Analysis Process
19	Reconstructing Drone Volatile Items
20	Recovering Drone Schema
21	Analysis of Drone Stages

**Table 5:** A comparative summary: the proposed CCAFM and existing drone forensic models.

ID	Processes in the Compared Models	Processes in the Proposed Model		
		Collection and Preservation	Reconstruction and Analysis	Post-Investigation
1.	Collecting Drone Data	✓	✓	×
2.	Drone Evidence Collection	✓	✓	×
3.	Collection Process	✓	✓	×
4.	Drone Items Collection	✓	✓	×
5.	Drone Data Extraction Process	✓	✓	×
6.	Starting the Investigation	✓	✓	×
7.	Drone Metadata Gathering	✓	✓	×
8.	Drone Data Collection	✓	✓	×
9.	Collecting Drone Files	✓	✓	×
10.	Drone Items Collection	✓	✓	×
11.	Drone Collection Process	✓	✓	×
12.	Collection of Drone Nonvolatile Items	✓	✓	×
13.	Collection of Drone Volatile Items	✓	✓	×
14.	Drone Items Collection	✓	✓	×
15.	Collection of Suspect Drone System	✓	✓	×
16.	Collection and Preservation	✓	✓	×
17.	Drone Collection Process	✓	✓	×
18.	Execution of Drone Data	✓	✓	×
19.	Collection, Preservation	✓	✓	×
20.	Drone Items Collection	✓	✓	×
21.	Drone Items Collection	✓	✓	×
22.	Reconstructing Drone Data	✓	✓	×
23.	Restoring Drone Integrity	✓	✓	×
24.	Drone Media Analysis	✓	✓	×
25.	Timeline Creation of Drone Data	✓	✓	×
26.	Drone Data Recovery	✓	✓	×
27.	Search String	✓	✓	×
28.	Drone Artifact Analysis	✓	✓	×
29.	Financial and Business Data Analysis	✓	✓	×
30.	Drone Restoration and Searchability	✓	✓	×
31.	Investigation on Drone Data Collected	✓	✓	×
32.	Drone Artifact Analysis	✓	✓	×
33.	Rebuilding of Drone Data	✓	✓	×
34.	Reconstruction of the Drone Events	✓	✓	×
35.	Drone Forensic Analysis	✓	✓	×
36.	Analysis of Anti-forensic Attacks, Analysis of Drone Attack	✓	✓	×
37.	Reconstructing Drone Evidence	✓	✓	×
38.	Reconstruction Drone Process	✓	✓	×
39.	Drone Analysis Process	✓	✓	×
40.	Reconstructing Drone Volatile Items	✓	✓	×
41.	Recovering Drone Schema	✓	✓	×
42.	Analysis of Drone Stages	✓	✓	×

## Data Availability

All the data used to support the study are included within the article.
